# Evaluation of Antioxidant and Cytotoxic Activity of Hydro-Ethanolic Extracts Obtained from *Steiractinia aspera* Cuatrec

**DOI:** 10.3390/molecules27134186

**Published:** 2022-06-29

**Authors:** Laura Gamboa-Carvajal, Carlos Jara-Gutiérrez, Joan Villena, Lautaro Taborga, Jairo René Martínez, Luis Espinoza, Elena E. Stashenko

**Affiliations:** 1 Departamento de Química, Universidad Técnica Federico Santa María, Avenida España 1680, Valparaíso 224000, Chile; laura.gamboa@sansano.usm.cl (L.G.-C.); lautaro.taborga@usm.cl (L.T.); 2 Laboratorio de Bioensayos, Centro de Investigaciones Biomédicas (CIB), Facultad de Medicina, Universidad de Valparaíso, Angamos 655, Reñaca, Viña del Mar 2340000, Chile; carlos.jara@uv.cl (C.J.-G.); juan.villena@uv.cl (J.V.); 3 Center for Chromatography and Mass Spectrometry CROM-MASS, Universidad Industrial de Santander, Bucaramanga 68000, Colombia; rene@tucan.uis.edu.co

**Keywords:** *Steiractinia aspera* Cuatrec, antioxidant capacity, ABTS on-line, PLC-DAD, cytotoxicity

## Abstract

In this work, the antioxidant activity of the hydro-ethanolic extracts of the leaves, flowers, and aerial parts of *Steiractinia aspera* Cuatrec, both fresh and post-distillation, was evaluated by ABTS^+·^, FRAP, H_2_O_2_ and DPPH assays. The cytotoxic activity was evaluated in MCF-7, MCF-10A and HT-29 cell lines. The hydro-ethanolic extracts were obtained by matrix solid-phase dispersion (MSPD) and ultrasound-assisted solvent extraction (SE). The fresh-leaf MSPD extract had the highest antioxidant activity, and the post-distillation leaf ultrasound-assisted SE extract had the highest cytotoxicity in the MCF-7 breast cancer cell line, although not selective, which was evaluated by sulforhodamine B assay. On the other hand, ROS was evaluated by flow cytometry which showed that post-distillation leaf extract is pro-oxidant. Chlorogenic acid, kaempferol-3-glucoside and quercetin were found in the fresh leaves’ extracts, according to HPLC-DAD. PLC-DAD permitted the isolation of *p*-coumaric acid, *E*-3-(4-(((*E*)-3-(3,4-dihydroxyphenyl) acryloyl) oxy)-3-hydroxyphenyl) acrylic acid and a diglucosylated derivative of ursolic acid, which were analyzed by ^1^H and ^13^C NMR. Our results suggest that the fresh leaf extract of *Steiractinia aspera* Cuatrec has potential use for antioxidant applications.

## 1. Introduction

The Asteraceae family has a cosmopolitan distribution and is one of the plant families with the greatest richness of plant species [1,2]. Among these is the species *Steiractinia aspera* Cuatrec, which is endemic and native to Colombia. This plant is one of the 50 promising species within the “Bio-Reto XXI 15:50” program. This program seeks to develop bioproducts for the health, agricultural and cosmetic sectors, as a result of the study of Colombian biodiversity.

For this species, no studies of the composition or biological activity have been reported, however eudesmanolide-like lactones were isolated from plants of the same genus, specifically *Steiractinia mollis* [3,4], which has confirmed anticancer and antioxidant activity [5,6,7,8].

Many of the sesquiterpene lactones and polyphenols have been shown to act as antioxidants [9] and anti-inflammatory agents [10].

Plant extracts are used in cosmetics to treat the skin, to beautify and maintain its physiological balance. In addition, they are biodegradable and have low toxicity, compared to the synthetic compounds used in the cosmetic sector [11]. In this context, due to the polyfunctional properties of the natural plant extracts, they can be used to create natural antioxidants, lighteners and topical preservatives [11].

On the other hand, the essential oil industry poses a problem of solid waste disposal, post distillation [12]. However, some of these by-products have potential as a source of bioactive compounds for skin application [11], which may represent the utilization of waste that is currently discarded, thus contributing to the circular economy.

This work examined the potential use of *Steriactinia aspera* as a source of extracts containing bioactive compounds, to be applied in the cosmetics sector as an antioxidant, together with a circular bioeconomy approach. The plant extracts were obtained by matrix solid phase dispersion (MSPD) and ultrasound-assisted solvent extraction (SE) from the vegetal material both before and after performing microwave-assisted hydrodistillation. The detailed characterization of these extracts aimed to detect the compositional changes that may result from the heat treatment during the hydrodistillation. HPLC-DAD, PLC-DAD, ^1^H and ^13^C NMR instrumental techniques were applied to extract the characterization. The assays used to evaluate the biological activities were: ABTS^+·^ radical sequestration by TRAP method; FRAP method; H_2_O_2_ sequestration; and DPPH radical sequestration, together with sulforhodamine B assay and ROS evaluation by flow cytometry. 

## 2. Results and Discussion

### 2.1. Extraction Yields

The non-volatile secondary metabolites present in the species *Steiractinia aspera* Cuatrec, in both fresh and post-distillation vegetal materials, were obtained by two techniques, ultrasound-assisted SE and MSPD, obtaining the yields presented in Table 1. The plant material collected was classified into leaves (H), flowers (F) and aerial parts (PA).

According to Table 1, a higher extraction yield was obtained by MSPD from the fresh than from the post-distillation plant material, because this technique combines aspects of several analytical extraction phenomena, that lead to disruption, dispersion and cleaning-up of the sample on a solid support. This changed the particle size, homogeneity and tissue integrity of the plant material. [13].

### 2.2. Phytoconstituents

All of the evaluated *Steiractinia aspera* Cuatrec organs had total phenol and flavonoid contents, as shown in Table 2.

According to the statistical method used, significant differences were found in two of the extracts of the fresh aerial parts obtained by ultrasound-assisted SE and MSPD. The hydro-ethanolic extract obtained by SE of the fresh aerial parts presented the highest concentration of total flavonoids (Table 2), while the extract obtained by MSPD from the fresh aerial parts presented a higher concentration of total phenols than the rest of the extracts (Table 2). As indicated above, these results led to the evaluation of antioxidant activity, since these compounds have a high capacity to scavenge for free radicals, donate hydrogen atoms or electrons, or to act as metal cation chelators [14]. 

### 2.3. Antioxidant Analysis

The total antioxidant capacity of each extract obtained by SE and MSPD was evaluated by two reaction mechanisms, single electron transfer (SET) and hydrogen atom transfer (HAT). Among these mechanisms, ABTS^+·^ radical sequestration assays, and ferric reducing antioxidant power (FRAP) assays were used for SET. By HAT, the tests were: H_2_O_2_ reduction and DPPH^·^ radical decolorization. The results reported in Table 3 were analyzed independently, because the assays have different mechanisms of action. The unit of measurement for the DPPH^·^ technique and the H_2_O_2_ sequestration technique was expressed in mean inhibitory concentration (IC_50_), therefore, the lower the value, the higher its reactive oxygen species (ROS) sequestration capacity.

According to the results presented in Table 3, the fresh leaves’ MSPD extract had significantly higher antioxidant capacity than all of the other extracts. On the other hand, the hydro-ethanolic extract of fresh flowers obtained by ultrasound-assisted SE showed significant differences and higher DPPH^·^ radical scavenging activity compared to the rest of the extracts (Table 3). In general, the antioxidant activity was higher in the extracts obtained from the fresh plant material than in the extracts from the post-distillation plant material, indicating that the temperature decreases the antioxidant capacity due to possible changes in the composition of the extract.

According to these results, the fresh plant material of the species studied has high potential as an antioxidant for industrial application. However, no extract exceeded the antioxidant capacity of the reference compounds used.

### 2.4. Identification of Phenolic Compounds by HLPC-DAD

**Figure 1:**
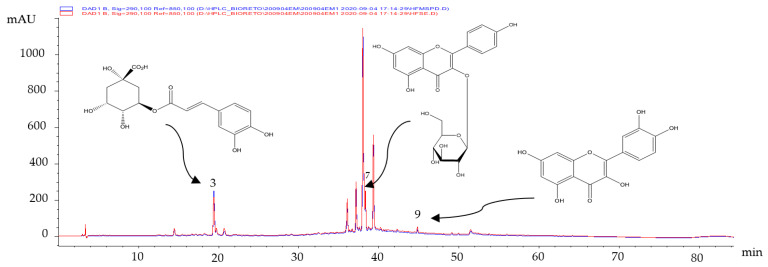
Chromatographic profiles obtained by HPLC-DAD of the fresh leaves’ MSPD extracts.

The secondary metabolites present in the extracts obtained from fresh leaves were identified by comparing their retention times (t_R_, min) and UV spectra with those of the certified standards. Three polyphenols (chlorogenic acid (**3**), Kaempferol-3-glucoside (**7**) and quercetin (**9**), respectively) were identified (Figure 1).

As shown in Figure 1, there were no differences in the composition of the hydro-ethanolic extract of fresh leaves obtained by the two extractive techniques employed. The identified compounds have presented multiple biological activities [15], such as antioxidant [16] and anticarcinogenic [17,18] activities. These compounds would be partly responsible for the results obtained in Table 3.

### 2.5. ABTS-Online

**Figure 2:**
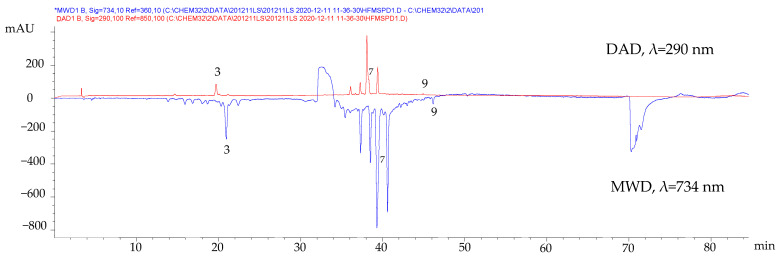
Chromatographic profiles of the fresh leaves MSPD extract before and after derivatization using ABTS reagent.

To confirm the antioxidant activity of the compounds identified by HPLC-DAD, the ABTS-online assay was performed. This test consisted of mixing the HPLC-DAD eluate with the ABTS^+·^ radical cation solution in a coil where the antioxidant species could react and cause an absorbance decrease, manifested as a negative peak in the ABTS signal at 734 nm monitored with a separate detector (multiple wavelength detector, MWD). In Figure 2, the negative peaks of compounds (**3**), (**7**), and (**9**) indicate their antioxidant nature. 

### 2.6. Cytotoxicity on Cancer Cell Lines

Based on the cell viability results, the mean inhibitory concentrations (IC_50_) of each extract and for each cell line (MCF-7 and MCF-10A) were calculated, and the cytotoxic activity was subsequently evaluated (Table 4).

According to the results in Table 4, the hydro-ethanolic extracts of the fresh plant material obtained by ultrasound-assisted SE showed a higher cytotoxicity on the MCF-7 breast cancer cell line than those obtained by MSPD. However, the cytotoxicity in those extracts also similarly affected the MCF-10A non-tumoral cell line, indicating that the cytotoxic activity is not selective. All of the hydro-ethanolic extracts were found to be non-cytotoxic against the HT-29 cell line.

Independent of the extraction technique, the post-distillation leaves showed cytotoxic activity. The difference in the results between the techniques employed indicates that the temperature could be causing a transformation or elimination of cytotoxic substances. Based on these results, the fresh plant material still has potential in cosmetic applications, due to its lack of cytotoxicity. 

On the other hand, one of the factors for cytotoxic non-selectivity is due to pro-oxidation. For this, ROS was evaluated by flow cytometry, considering the IC_50_ of the hydro-ethanolic extract with higher cytotoxicity. (post-distillation leaves obtained by ultrasound-assisted SE).

### 2.7. Evaluation of ROS Production by Flow Cytometry

ROS production was evaluated in the hydro-ethanolic extract of the post-distillation leaves (E4) (IC_50_ concentration). The treatment was performed for 24 and 48 h, using DCFH_2_-DA to evaluate the production of these reactive species. Solvent control (CS) and a positive control (C+) of daunorubicin (DRN) were used.

**Figure 3:**
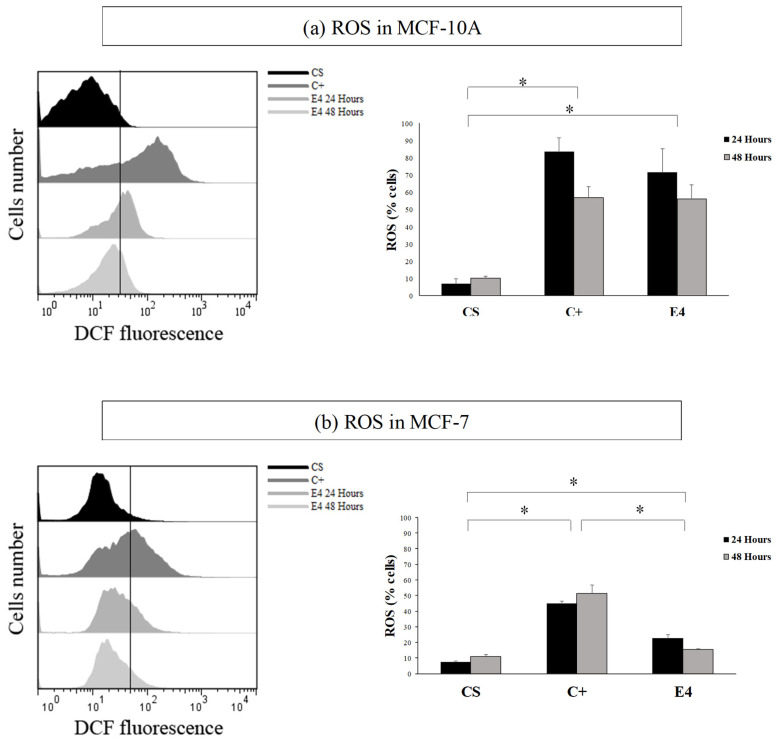
Comparative histogram between cell number and DCFH_2_-DA fluorescence, on lines (**a**) MCF-10A and (**b**) in MCF-7. CS = solvent control; C+ = positive control; E4 = post-distillation hydro-ethanolic leaf extract obtained by ultrasound-assisted SE. Data are expressed as mean percentages ± standard deviation (*n* = 3). * Corresponds to significant differences between treatments per cell line, (*p* < 0.05).

The histogram in Figure 3 showed that, in both the MCF-7 and MCF-10A cell lines, the treatment increased the fluorescence in the cells with respect to the control (in blue). This effect was not selective, in a similar way as the data of cytotoxicity. The highest fluorescence in both of the cases was observed at 24 h with respect to C+ (red).

With the result obtained in Figure 3, it was possible to visualize that, after treatment, ROS was produced in both of the cell lines (which could generate oxidative damage). This result indicated that the post-distillation leaf extract is pro-oxidant. In this context, this extract could have potential applications.

### 2.8. Fractionation and Isolation of Phenolic Compounds

The solvents of different polarity were employed in ultrasound-assisted solid-liquid exhaustive extractions of fresh leaves (50 g) followed by liquid–liquid fractionation. Table 5 presents the yields and extract amounts obtained in the various extractions performed. 

From the aqueous fraction, as a result, three of the compounds were isolated, using an acidified water ratio of 86.7 and 60%, respectively. 

### 2.9. Compound Identification by Spectroscopic Techniques

Based on the fractions obtained by preparative HPLC, three major compounds were tentatively identified by NMR 1D (Figure S1), NMR 2D (Figure S2) and by IR and MS (Figure S3), where compound (**a**) corresponded to p-coumaric acid. The signals in the spectrum NMR coincided with those reported in the literature [19]. 

The compound (**b**) corresponded to the (*E*)-3-(4-(((*E*)-3-(3,4-dihydroxyphenyl)acryloyl)oxy)-3-hydroxyphenyl) acrylic acid. The signals in the spectrum NMR coincided with those reported in the literature [20]. The identification was performed by NMR 1D (Figure S4), NMR 2D (Figure S5) and by IR (Figure S6).

Finally, the compound (**c**) was a diglucosylated derivative of ursolic acid, where the signals in the spectrum NMR coincided with those reported in the literature [21,22,23]. In the same way, the identification was carried out by NMR 1D (Figure S7), NMR 2D (Figure S8) and by IR (Figure S9).

The *p*-coumaric acid and (*E*)-3-(4-(((*E*)-3-(3,4-dihydroxyphenyl)acryloyl)oxy)-3-hydroxyphenyl)acrylic acid, are hydroxycinnamic acids with reported antioxidant, anticancer and pro-oxidant activity [24]. From diglucosylated ursolic acid, which is a pentacyclic triterpene, the antioxidant, antimicrobial, anti-inflammatory and anti-carcinogenic activity have been evidenced [25]. These results, together with the compounds identified by HPLC-DAD, would explain the antioxidant activity reported in Table 3.

## 3. Materials and Methods

### 3.1. Reagents

The reagents and solvents used in the HPLC-DAD and the antioxidant activity assays were of analytical grade, obtained from Sigma-Aldrich (St. Louis, MO, USA).

In the cytotoxic and anticancer tests, the DMEM medium Invitrogen (Los Angeles, CA, USA), absolute ethanol from Sigma-Aldrich (St. Louis, MO, USA) and the colon (HT-29), breast (MCF-10A) and cancerous breast (MCF-7) cell lines, obtained from the Type Culture Collection (ATCC) (Rockville, MD, USA), were used.

For the isolation and purification of the compounds present in the hydro-ethanolic extract of the fresh leaves, type I water and acetonitrile HPLC preparative grade, acquired from Merck (Darmstadt, Germany), were used as elution solvents. The aqueous extract was pre-cleaned with commercial grade hexane, dichloromethane and ethyl acetate.

### 3.2. Plant Material

The species studied in this work was collected in the city of Bucaramanga, Colombia, in January 2020, under the contract for access to genetic resources and derived products for bioprospecting purposes N° 270, between the Ministry of Environment and Sustainable Development and Universidad Industrial de Santander.

This species was propagated at the Pilot Agroindustrial Complex of the National Research Center for the Agroindustrialization of Aromatic and Medicinal Tropical Plant Species (CENIVAM), at the main campus of the Universidad Industrial de Santander, (977 masl, N 07° 08,422′ W 073° 06,960′) (Bucaramanga, Colombia). The taxonomic identification was performed in the Herbarium of the University, voucher N° 20,891 (Table S1).

From this plant, the fresh leaves, flowers and aerial parts were analyzed separately, along with post-distillation leaves and aerial parts from which the non-volatile secondary metabolites were obtained by different extractive techniques.

### 3.3. Extraction Techniques

#### 3.3.1. Solid-Phase Matrix Dispersion Extraction (MSPD)

The hydro-ethanolic extract was obtained by the MSPD method using the methodology proposed by Xiao et al. [26], where 0.5 g of the dried, ground and sieved plant material was mixed with 2 g of silica-C18 as the dispersing agent. The mixture was macerated and the polar compounds were eluted with an ethanol: water mixture (70:30 *v*/*v*). The solvent was removed from the extract obtained by a stream of nitrogen gas and the water was subsequently removed by freeze-drying.

#### 3.3.2. Ultrasound-Assisted Solvent Extraction (SE)

The solvent extraction was carried out according to Londoño-Londoño et al. [27], with some modifications.

The dried plant material, ground and sieved, was deposited (1 g) in an amber-colored flask (60 mL) and an ethanol: water solution type I (70:30 *v*/*v*) was added. The suspension was placed in an ultrasonic bath (Elmasonic S15H, Singen, Germany) for 1 h at 50 °C. The residue was filtered, and the solvent was roto-evaporated. The hydro-ethanolic extracts obtained by SE and MSPD were dried in a lyophilizer (VirTis AdVantage Plus) for 48 h and stored at 4 °C in the absence of light. Subsequently, the extraction yields were calculated, and the respective chromatographic analyses were performed, together with the evaluation of the antioxidant activity. Each extraction was performed in triplicate.

### 3.4. Analysis of Phytoconstituents

Polyphenols have been linked to a wide range of biological properties, such as antioxidant, antifungal, antibacterial, anti-inflammatory and anti-carcinogenic activities [28]. Based on the above, in this research the content of the total phenols and flavonoids as phytoconstituents present in the species *Steiractinia aspera* Cuatrec was determined to evaluate its antioxidant activity.

#### 3.4.1. Determination of Total Phenols

The determination of the total phenol content was carried out, using the spectrophotometric methodology proposed by Waterman and Mole, 1994 [29]. In this methodology, the Folin–Ciocalteu reagent and gallic acid, as a reference standard, were used. The result of the total phenol content was expressed in mg gallic acid/g dry weight from a calibration curve. In this assay, 250 µL of each hydro-ethanolic extract obtained by both ultrasound-assisted SE and MSPD (1 mg/mL) were taken and mixed with 1.25 mL of Folin–Ciocalteu reagent (0.2 N). This mixture was incubated for 5 min at room temperature and then 1 mL of Na_2_CO_3_ (7.5%) was added to obtain the basic medium. Finally, the mixture was incubated for 2 h. The absorbances were measured in the spectrophotometer at a wavelength of 700 nm. Each extract was analyzed in triplicate.

#### 3.4.2. Determination of Total Flavonoids

The total flavonoids were determined spectrophotometrically, since this type of compound reacts by forming a colored chelate when in contact with aluminum chloride (AlCl_3_). For this, 1 mL of each hydro-ethanolic extract obtained by both ultrasound-assisted SE and MSPD was taken at a concentration of 1 mg/mL and 1 mL of a solution of AlCl_3_ (2% in ethanol) was added to it. After this, the samples were incubated at room temperature for 15 min. The absorbances were measured in the spectrophotometer at a wavelength of 430 nm. Each extract was analyzed in triplicate and the results were expressed in mg quercetin (QE)/g dry weight, based on a calibration curve [30].

### 3.5. Assessment of Antioxidant Activity by Different Action Mechanisms 

The in vitro methods used for the evaluation of antioxidant activity in this research were: sequestration of the ABTS^+·^ radical by the TRAP method; the FRAP method; sequestration of H_2_O_2_; and scavenging of the DPPH radical [31,32]. 

#### 3.5.1. Total Reactive Antioxidant Properties Assay

In this assay, the methodology of Romay et al. (1996) [33] was used, where the stable radical 2,2′-azinobis-(3-ethylbenzothiazolin-6-sulfonic acid) (ABTS), whose coloration is intense blue-green, decreased in the presence of antioxidants, obtaining as a response a decrease in the absorbance measured in the spectrophotometer. Experimentally, 10 μL of each extract (1 mg/mL) was taken and mixed with 990 μL of the ABTS^+·^ cation-radical solution (ABTS 150 μM in PBS at pH 7.4 and ABAP 10 mM solution), to finally measure the absorbance at 734 nm, in periods of 50 s. The blank was performed with ABTS^+·^ solution. The radical inhibition percentage was calculated, using Equation (1):

(1) $$IR,\;\left(\%\right)=\left(\frac{\left(A_{0}-A_{50}\right)}{A_{0}}\right)\times 100$$

where *A*_0_ is the absorbance at time 0 s and *A*_50_, the absorbance at 50 s. The results were extrapolated in a calibration curve with Trolox^®^ and expressed in μmolTrolox^®^/g dry weight where, subsequently, these were compared with the reference standards (gallic acid and butylated hydroxytoluene (BHT)). Each extract was evaluated in triplicate.

#### 3.5.2. Ferric Reducing Antioxidant Potential (FRAP) Assay

This assay was based on the methodology of Dudonné et al. (2009) [34], where 100 μL of each hydro-ethanolic extract (1 mg/mL) was taken and mixed with 300 μL of type I water and 3 mL of FRAP solution (10 volumes of 300 mM acetate buffer, 1 volume of 20 mM FeCl_3_ and 1 volume of 10 mM TPTZ (2,4,6-tri(2-pyridyl)-s-triazine)). The mixture was shaken and incubated for 30 min in a thermoregulated bath at 37 °C. After this time, the absorbance was measured at 593 nm, extrapolating the results on a Trolox^®^ calibration curve and expressing them in μmolTrolox^®^/g dry weight where, subsequently, these were compared with the reference standards (gallic acid and butyl-hydroxytoluene (BHT)). The blank was performed with absolute ethanol. Each extract was evaluated in triplicate.

#### 3.5.3. Hydrogen Peroxide Scavenging Activity

According to the methodology of Ruch et al. (1989) [35], the effect of each hydro-ethanolic extract as hydrogen peroxide (H_2_O_2_) reductants was evaluated. This evaluation was performed at three concentrations (0.5, 2.0 and 3.5) mg/mL. To determine the above effect, 5 μL of each extract was taken and 2.9 mL of type I water and 100 μL of H_2_O_2_ solution (H_2_O_2_ 40 mM in 50 mM phosphate buffer at pH 7.4) were added. The mixture was shaken and incubated for 10 min at room temperature, where the absorbance was subsequently measured at 240 nm. The blank was performed with type I water. The percentage inhibition of the H_2_O_2_ radical (*IR*, %) was calculated, using Equation (2). 

From each result obtained, the mean inhibitory concentration (IC_50_, mg/mL), was calculated and compared with two reference antioxidants, Trolox^®^ and BHT.

(2) $$IR,\;\left(\%\right)=\left(\frac{\left(A_{Control}-A_{Sample}\right)}{A_{Control}}\right)\times 100$$

where *A_Control_* is the absorbance of H_2_O_2_ without the sample and *A_Sample_* is the absorbance of the H_2_O_2_ solution with the extract.

#### 3.5.4. DPPH Radical Scavenging Assay

Based on the methodology of Brand-Williams et al. (1995) [36], the radical DPPH (2,2′-dinitrophenyl-1-picrylhydrazyl) was used to evaluate the reducing capacity of each hydro-ethanolic extract. For this, 100 μL of the extract was mixed with 2.9 mL of a solution of DPPH 50 μM in ethanol. The mixture was shaken and incubated for 15 min at room temperature to finally measure the absorbance at 517 nm. This assay was performed in triplicate at three concentrations of each extract (1.0, 2.5 and 5.0) mg/mL. The control and blank were performed with absolute ethanol. With the absorbance obtained, the percentage inhibition of the DPPH radical was calculated, (IR, %), by means of Equation (2).

### 3.6. Analysis of Phenolic Compounds by HLPC-DAD

The extract with the highest antioxidant activity was analyzed on a high-performance liquid chromatograph coupled to a diode array detector, HPLC-DAD, Agilent Technologies (Palo Alto, CA, USA). The separation of the compounds was performed on a GEMINI C18 column (Phenomenex, Torrance, CA, USA), 250 mm, L × 4.6 mm, I.D. × 5 µm particle size, 25 °C, using as mobile phase (A): H_2_O (0.5% CH_2_O_2_) and (B): acetonitrile, at a flow rate of 1 mL/min, with an elution gradient 0 min: 98% A, 15 min: 88% A, 15–23 min: 88% A, 46 min: 60% A, 71 min: 10% A, 71 min: 10% A, 71–75 min: 10% A, 80 min: 98% A, 80–85 min: 98% A. The injection volume was 20 µL and the phenolic compounds were detected at 290 nm. For the identification of the phenolic compounds, 13 certified standards were used (Figure S10).

### 3.7. ABTS On-Line

The extract with the highest antioxidant activity was analyzed in a high-performance liquid chromatograph HPLC, consisting of a diode array detector (DAD) and a multi-wavelength detector (MWD), all from Agilent Technologies (Palo Alto, CA, USA). The HPLC-DAD was coupled to a Pinnacle PCX post-column derivatizer from Pickering Laboratories (Mountain View, CA, USA). The analytical column and chromatographic conditions were the same as those used in the HPLC-DAD analysis, as indicated in Section 3.6. For this assay, a methodology developed at CENIVAM (UIS-Colombia) was used, following the procedure of Koleva et al. (2001) [37], with some modifications.

According to the results reported by Kusznierewicz et al. (2011) [38], a stock solution of ABTS^+·^ (7 mM) was prepared with type I water and mixed with an aqueous solution of potassium persulfate (2.45 mM). The mixture was incubated for 16 h in the dark at room temperature. From the stock solution, the working solution (210 µM) was prepared, the absorbance of which was adjusted to 0.710 ± 0.003, and then introduced into the reactor with a storage time of no more than 48 h. The temperature in the reactor was at 130 °C, and the flow rate of the derivatizer was 0.5 mL/min, thus maintaining a concentration of ABTS^+·^ in the reactor of 70 µM. Absorbance was measured at three wavelengths: 515 nm (maximum absorption of anthocyanins), 290 nm (maximum absorption of other phenolic compounds) and 734 nm (absorption of the ABTS^+·^ radical cation) [39]. 

### 3.8. Evaluation of Cytotoxic Activity

The antioxidant activity of the phenolic compounds present in plants has been linked to a decrease in the risk of developing cancer [40]. Based on this, the cytotoxicity of the hydro-ethanolic extracts was evaluated by initially preparing a cell culture and performing the sulforhodamine B cytotoxicity assay, and finally the evaluation of the reactive oxygen species (ROS) by flow cytometry.

#### 3.8.1. Cell Culture

In this research, two human cancer cell lines and a normal non-cancerous control line were used: colon cancer line HT-29 (human colorectal adenocarcinoma); breast cancer line MCF-7 (human mammary gland adenocarcinoma); and normal breast epithelial cell line MCF-10A. The medium used for the cell culture was DMEM containing: 10% fetal bovine serum; penicillin (100 U/mL); streptomycin (100 µg/mL); and glutamine (1 mM). Prior to the addition of the extracts, the cells were seeded in the medium using 96-well cell culture plates. The cells were then treated with the extracts for 72 h at 37 °C in a 5% carbon dioxide (CO_2_) atmosphere with 95% humidity.

#### 3.8.2. Sulforhodamine B Cytotoxicity Assay

Using the methodology of Skehan et al. (1990) [41], the cytotoxicity was measured through the cell density. This process consisted of an intense pink aminoxanthene staining, which indicated that the sulforhodamine B was bound to the proteins of the cultured cells (previously fixed with trichloroacetic acid). The amount of this dye extracted is directly proportional to the cell mass. Based on this, experimentally, in 96-well plates at a density of 5 × 10^3^ cells/well, 100 μL of medium was added, on which the cell lines were seeded. These cells were incubated for 24 h, after which the 10 hydro-ethanolic extracts (five obtained by MSPD and five obtained by ultrasound-assisted SE of different plant organs) were added at different concentrations (25, 50 and 100) µg/mL. As a negative control (C-), the cell culture without addition of the sample was used, and the cell count was performed as a reference of the logarithmic growth at 24 h. The solvent control (CS) received only the medium containing 1% ethanol with which the samples were solubilized.

After the incubation period, the cells were fixed with 25 µL trichloroacetic acid (50%) for 1 h at 4 °C and washed with distilled water. The staining was performed with 50 µL/well of sulforhodamine B, incubated again for 30 min at room temperature and then washed with 150 μL/well of 1% acetic acid and dried at room temperature. Finally, the dye was solubilized with 100 μL/well of 10 mM Tris Base. The absorbance was measured at 540 nm. The percentage viability was calculated, using Equation (3), which indicates cell survival after treatment with all extracts:

(3) $$Viability,\;\%=\frac{A_{Sample}}{A_{Control}}\times 100$$

where *A_Sample_* is the absorbance obtained from each extract and *A_Control_* is the absorbance of the solvent control. All of the assays were performed in triplicate.

#### 3.8.3. Evaluation of ROS by Flow Cytometry

The flow cytometry test was performed on the extract with the highest cytotoxic activity, using the methodology of Rothe and Valet (1990) [42]. Accordingly, 2′-7′-dichlorodihydrofluorescein diacetate (DCFH_2_-DA) was used for the determination of oxidative stress. This compound, when deacetylated by cytosolic esterase enzymes, loses fluorescence when transformed into 2′-7′-dichlorodihydrofluorescein (DCFH_2_), which in the presence of ROS is oxidized at two sites of its structure, becoming 2′-7′-dichlorofluorescein (DCF), which is a fluorescent compound. In this test, the fluorescence inside the cells is proportional to the amount of ROS produced.

Experimentally, in a 6-well plate, the treatments were a positive control (C+) that generated ROS, in this case corresponding to daunorubicin (DNR) 1 μM, absolute ethanol was used as the solvent control (CS) and the extract evaluated at its IC_50_. The treatments were applied to the culture every 24 and 48 h and remained incubated at 37 °C. After this time, the cells were labeled with 10 μL per well of 1 mM DCFH_2_-DA and incubated for 1 h at 37 °C. Subsequently, the medium was removed and washed three times with 1000 μL of phosphate-buffered saline (PBS, 1.5 M NaCl, 20.1 mM KH_2_PO_4_ and 63.7 mM Na_2_HPO_4_, adjusted to pH 7.4). 

To each well, 200 μL of trypsin was added, in order to detach the adherent cells, incubating them for 10 min at 37 °C. Continuing with the process, 300 μL of cell medium was added as a blocking agent and the contents were transferred into 1.5 mL tubes and centrifuged for 10 min at 1500× *g*, where the supernatant was removed and the pellet was re-suspended in 500 μL of PBS. Finally, the emitted fluorescence was measured via flow cytometry using the FL1 filter. The data obtained were plotted in histograms using FlowJo 7.6.2 software and expressed as a percentage of the cells positive for DCF fluorescence. 

### 3.9. Fractionation and Isolation of Phenolic Compounds 

The hydro-ethanolic extract with the highest antioxidant activity was previously fractionated, as described in the flow diagram in Figure S11. At each stage of the fractionation, the organic solvent was removed by roto-evaporation, thus allowing the calculation of the extraction yield.

The isolation and purification of the major phenolic compounds present in the plant organ with the highest antioxidant activity were carried out from the aqueous extract obtained by the fractionation previously performed. For this isolation, preparative liquid chromatography coupled to a Gilson PLC 2250 diode array detector (PLC-DAD) was used. The separation of the compounds was performed on a SiliaChrom Plus C18 column (2500 Parc-Technologique blvd, Quebec City, QC, Canada), 250 mm, L × 50 mm, I.D. × 10 µm particle size, 100 Å pore size, at room temperature and a maximum pressure of 200 Bars. For the mobile phase (A): H_2_O (0.5% CH_2_O_2_) and (B): acetonitrile, at a flow rate of 60 mL/min, with an elution gradient: 2 min: 98% A, 7 min: 88% A, 7–23 min: 88% A, 23–70 min: 60% A, 70–72 min: 60% A, 72–80 min: 10% A, 80–82 min: 10% A, 82–87 min: 98% A, 87–89 min: 98% A, were used.

From the aqueous extract, 700 mg of sample was weighed and made up to 10 mL with type I water. The sample was filtered through a 0.45 µm PTFE disc and injected into the 10 mL PLC-DAD loop.

The chromatographic conditions at the preparative level were scaled, based on the conditions used in the analytical chromatography (HPLC-DAD), according to the equations recommended by the Waters^™^ supplier [43]. 

The compound identification was done in DAD scan mode, collecting each compound in different test tubes where the solvent was subsequently removed by roto-evaporation to calculate the extraction yields. 

### 3.10. Identification of Compounds by NMR

The ^1^H and ^13^C NMR spectra were recorded in deuterated methanol (MeOD) solutions and were referenced to the residual peaks of CD_3_OD at δ = 3.30 ppm and δ = 49.00 ppm for ^1^H and ^13^C, respectively, on an Avance Neo 400 Digital NMR spectrometer (Bruker, Rheinstetten, Germany) operating at 400.1 MHz for ^1^H and 100.6 MHz for ^13^C.

### 3.11. Statistical Analysis

In this work, for the parametric and nonparametric data (defined by distribution tests), one-way ANOVA and Kruskal–Wallis ANOVA were performed, respectively. A *p* < 0.05 was considered a significant difference and the results were expressed as (mean ± standard deviation (SD). The software used was STATISTICA 7.0.

## 4. Conclusions

According to the extraction techniques used, a higher extraction yield was observed with the MSPD than with the ultrasound-assisted SE. As for the identification of the phenolic compounds by HPLC-DAD, there were no differences between them. Therefore, the use of each of them will depend on the objective of further studies on this species.

The cytotoxic activity was evidenced in all of the extracts obtained by ultrasound-assisted SE, and in the post-distillation leaf extract obtained by the MSPD. This fact could be attributed to the effect of the temperature applied in the extraction methodology, that could be causing an elimination or transformation of the compounds from the plant matrix. The extract is not anti-carcinogenic due to the non-selectivity in the assays performed. Based on this, the generation of the ROS was evaluated in the cell lines where it was found that it presented pro-oxidation. In relation to this, future works could identify the compounds present in the distillation residue which cause high oxidative damage.

Regarding the focus of this research, it was found that the species *Steiractinia aspera* Cuatrec is a source of bioactive substances with potential application in the cosmetic industry, due to its antioxidant activity and the absence of cytotoxicity in the fresh plant material. In addition to this, the post-distillation plant material could become a valuable waste by taking advantage of its pro-oxidant properties, thus contributing to the circular economy.

## Figures and Tables

**Table 1:** Extraction yields of fresh and post-distillation plant material with the different extraction techniques used.

Plant Material	Extraction Yields, % ± s (*n* = 3)			
	SE		MSPD	
	Fresh	Post-Distillation	Fresh	Post-Distillation
H	11.7 ± 0.3	6.6 ± 0.35	15.4 ± 0.2	6.6 ± 0.4
F	8.2 ± 0.3	-	10 ± 1.4	3.1 ± 0.5
PA	9.4 ± 0.8	7.0 ± 0.8	13.7 ± 0.5	8.4 ± 0.3

^-^ Not realized.

**Table 2:** Concentration of phytoconstituents present in the extracts obtained by different extraction techniques, according to plant material state and plant part.

Extraction Technique	Condition of Plant Material	Plant Material	Total Phenols	Total Flavonoids
			(mg GAE/g Dry Weight)	(mg QE/g Dry Weight)
SE	Fresh	H	127 ± 7.9 ^a^	30 ± 2.9 ^a^
		F	129 ± 6.8 ^a^	21.0 ± 0.8 ^a^
		PA	128 ± 1.7 ^a^	31 ± 1.3 ^ab^
	Post-distillation	H	61.9 ± 0.3 ^ab^	21.3 ± 0.9 ^a^
		PA	79 ± 4.1 ^a^	18 ± 1.2 ^a^
MSPD	Fresh	H	135 ± 5.1 ^a^	29.7 ± 0.9 ^a^
		F	117 ± 3.9 ^a^	17.9 ± 0.9 ^a^
		PA	141 ± 2.8 ^ac^	27 ± 3.1 ^a^
	Post-distillation	H	66 ± 1.9 ^ab^	14.9 ± 0.8 ^ac^
		PA	89 ± 2.7 ^a^	16.6 ± 0.9 ^a^

^a–c^ Different letters (super indices) correspond to significant differences between extracts by phytoconstituent (*p* < 0.05). All data are expressed as mean ± s (*n* = 3). Total phenol content is expressed as mg of gallic acid equivalents (GAE) per gram of dry plant material weight. Total flavonoid content is expressed as mg quercetin equivalents (QE) per gram dry weight. H = Leaves; F = Flowers; PA = Aerial parts; SE = Ultrasound-assisted solvent extraction; MSPD = Matrix solid phase dispersion.

**Table 3:** Total antioxidant capacity for the different extracts obtained by different extraction techniques, plant material and plant part condition.

Extraction Technique	Condition of Plant Material	Plant Material	Antioxidant Assay, (Mechanism)			
			ABTS (SET)	FRAP (SET)	H_2_O_2_ (HAT)	DPPH (HAT)
			(μmolTrolox^®^/g Dry Weight)	(μmolTrolox^®^/g Dry Weight)	(IC_50,_ mg/mL)	(IC_50,_ mg/mL)
SE	Fresh	H	64 ± 1.4 ^a^	1290 ± 27 ^a^	7 ± 1.9 ^a^	0.67 ± 0.01 ^a,b^
		F	112 ± 3.9 ^a^	1320 ± 39 ^a^	4,8 ± 0.3 ^a,b^	0.63 ± 0.02 ^a,b,d^
		PA	130 ± 7.2 ^a^	1320 ± 56 ^a^	7,2 ± 0.3 ^a^	0.94 ± 0.01 ^a^
	Post-distillation	H	44 ± 1.6 ^a^	1300 ± 61 ^a^	7 ± 2.5 ^a^	3.42 ± 0.077 ^a,b,c,e^
		PA	28 ± 1.14 ^a,b^	1030 ± 43 ^a^	6 ± 1.1 ^ab^	2.87 ± 0.032 ^a^
MSPD	Fresh	H	147 ± 9.3 ^ac^	1340 ± 51 ^a^	4,5 ± 0.4 ^a,b,d^	0.81 ± 0.01 ^a^
		F	97 ± 2.3 ^a^	1310 ± 42 ^a^	8 ± 1.2 ^a^	0.790 ± 0.005 ^a^
		PA	109 ± 2.5 ^a^	1320 ± 51 ^a^	8 ± 1.2 ^a^	0.84 ± 0.01 ^a^
	Post-distillation	H	22 ± 10.7 ^a,b^	1240 ± 33 ^a^	8,6 ± 0.9 ^a,b,c,e^	4.13 ± 0.13 ^a,c^
		PA	52 ± 1.7 ^a^	1290 ± 44 ^a^	10 ± 2.1 ^a,c^	1.86 ± 0.02 ^a^
Reference compounds		Gallic Acid	1130 ± 10 ^d^	1720 ± 20 ^b^	N.A	N.A
		BHT	1060 ± 20 ^e^	1520 ± 70 ^c^	2.53 ± 0.06 ^f^	0.060 ± 0.0001 ^f^
		TROLOX^®^	N.A	N.A	2.86 ± 0.03 ^g^	0.1067 ± 0.0057 ^g^

^a–g^ Different letters (super indices) correspond to significant differences between extracts by total antioxidant capacity analysis (*p* < 0.05). All data are expressed as mean ± s (*n* = 3). ABTS radical scavenging and ferric reducing antioxidant power (FRAP) are expressed in μmol of Trolox^®^ equivalents per g dry weight. DPPH radical and H_2_O_2_ sequestration are expressed in mg/mL of mean inhibitory concentration (IC_50_). SET = Single electron transfer; HAT = Hydrogen atom transfer; BHT = Butylated hydroxytoluene; N.A. = Not applicable; H = Leaves; F = Flowers; PA = Aerial parts; SE = Solvent extraction assisted by ultrasound; MSPD = Matrix solid phase dispersion.

**Table 4:** Cytotoxic activity of extracts on HT-29, MCF-7 and MCF-10A cell lines.

Extraction Technique	Condition of Plant Material	Plant Material	Cell Line, (IC_50_, µg/mL)		
			HT-29	MCF-7	MCF-10A
SE	Fresh	H	>120 ^b^	87 ± 9.1 ^a^	80 ± 6.1 ^a^
		F	>120 ^b^	>120 ^b^	114 ± 2.4 ^a^
		PA	>120 ^b^	115 ± 17 ^a^	92 ± 8.3 ^a^
	Post-distillation	H	>120 ^b^	80 ± 11 ^a^	79 ± 1.2 ^a^
		PA	>120 ^b^	88 ± 17 ^a^	83 ± 4.0 ^a^
MSPD	Fresh	H	>120 ^b^	>120 ^b^	>120 ^b^
		F	>120 ^b^	>120 ^b^	>120 ^b^
		PA	>120 ^b^	>120 ^b^	>120 ^b^
	Post-distillation	H	>120 ^b^	90 ± 17 ^a^	88 ± 8.7 ^a^
		PA	>120 ^b^	>120 ^b^	>120 ^b^

^a,b^ Different letters (superindices) correspond to significant differences between extracts by cytotoxic activity analysis (*p* < 0.05). All data are expressed as mean ± s (*n* = 3). H = Leaves; F = Flowers; PA = Aerial parts; SE = Ultrasound-assisted solvent extraction; MSPD = Matrix solid phase dispersion.

**Table 5:** Ultrasound-assisted exhaustive extraction with different solvents of fresh leaves of *Steiractinia aspera* C.

Plant Material	Solvent	Solid–Liquid Extraction		Liquid–Liquid Extraction	
		Extract, g	Yield, %	Extract, g	Yield, %
Fresh leaves	Ethanol:water	9.13	1823	6.11	-
	Hexane	0.47	0.94	0.54	8.84
	Dichloromethane	0.32	0.64	0.59	9.65
	Ethyl acetate	0.08	0.17	0.95	15.46
	Water	-	-	3.89	63.58

## Data Availability

The data presented in this study are available in the Supplementary Material.

## Supplementary Materials

The following are available online at https://www.mdpi.com/article/10.3390/molecules27134186/s1, Figure S1. Spectra obtained by ^1^H NMR (400.144 MHz, CD_3_OD) and by ^13^C NMR (100.626 MHz, CD_3_OD) of the compound (a): *p*-coumaric acid; Figure S2. 2D NMR Spectra HSQC and HMBC of the compound (a): *p*-coumaric acid; Figure S3. IR spectrum and Mass spectra of (a): *p*-coumaric acid (HCD, 10 eV), obtained by UHPLC-ESI^+^-Orbitrap-MS; Figure S4. Spectra obtained by ^1^H NMR (400.144 MHz, CD_3_OD) and by ^13^C NMR (100.626 MHz, CD_3_OD) of the compound (b): (*E*)-3-(4-(((*E*)-3-(3,4-dihydroxyphenyl)acryloyl)oxy)-3-hydroxyphenyl)acrylic acid, respectively; Figure S5. 2D NMR Spectra HSQC and HMBC of the compound (b): (*E*)-3-(4-(((*E*)-3-(3,4-dihydroxyphenyl)acryloyl)oxy)-3-hydroxyphenyl)acrylic acid; Figure S6. IR spectrum of the compound (b): (*E*)-3-(4-(((*E*)-3-(3,4-dihydroxyphenyl)acryloyl)oxy)-3-hydroxyphenyl)acrylic acid; Figure S7. Spectra obtained by ^1^H NMR (400.144 MHz, CD_3_OD), ^13^C NMR (100.626 MHz, CD_3_OD) and Dept-135 of the compound (c): diglucosylated ursolic acid; Figure S8. 2D NMR Spectra HSQC and HMBC of the compound (b): diglucosylated ursolic acid; Figure S9. IR spectrum of the compound (b): diglucosylated ursolic acid; Table S1. Taxonomic identification; Figure S10. Chromatographic profile and retention times obtained by HPLC-DAD of certified reference phenolic compounds; Figure S11. Ultrasound-assisted, exhaustive extraction steps. In purple, liquid-liquid extraction. In green, solid-liquid extraction.

## Sample Availability

Samples of the compounds are not available from the authors.
