# Supplementary material for: Evaluation of Antioxidant and Cytotoxic Activity of Hydro-Ethanolic Extracts Obtained from Steiractinia aspera Cuatrec
Source: Molecules. 2022 Jun 29;27(13):4186. doi: 10.3390/molecules27134186 (PMC9268250; doi:10.3390/molecules27134186)
Supplement: Supplementary file 1 [file molecules-27-04186-s001.zip › molecules-1772759-supplementary.pdf]

# Evaluation of Antioxidant and Cytotoxic Activity of Hydro-Ethanolic Extracts Obtained from *Steiractinia aspera* Cuatrec

**Laura Gamboa-Carvajal [1], Carlos Jara-Gutiérrez [2], Joan Villena [2], Lautaro Taborga [1], Jairo René Martínez [3], Luis Espinoza [1,*] and Elena E. Stashenko [3,*]**

1 Departamento de Química, Universidad Técnica Federico Santa María, Avenida España 1680, Valparaíso 224000, Chile; laura.gamboa@sansano.usm.cl (L.G.-C.); lautaro.taborga@usm.cl (L.T.)
2 Laboratorio de Bioensayos, Centro de Investigaciones Biomédicas (CIB), Facultad de Medicina, Universidad de Valparaíso, Angamos 655, Reñaca, Viña del Mar 2340000, Chile; carlos.jara@uv.cl (C.J.-G.); juan.villena@uv.cl (J.V.)
3 Center for Chromatography and Mass Spectrometry CROM-MASS, Universidad Industrial de Santander, Bucaramanga 68000, Colombia; rene@tucan.uis.edu.co
* Correspondence: luis.espinozac@usm.cl (L.E.); elena@tucan.uis.edu.co (E.E.S.)

**Supplementary Materials**: The following are available online at www.mdpi.com/xxx/s1, **Figure S1**. Spectra obtained by $^1$H NMR (400.144 MHz, $CD_3OD$) and by $^{13}$C NMR (100.626 MHz, $CD_3OD$) of the compound (a): *p*-coumaric acid; **Figure S2**. 2D NMR Spectra HSQC and HMBC of the compound (a): *p*-coumaric acid; **Figure S3**. IR spectrum and Mass spectra of (a): *p*-coumaric acid (HCD, 10 eV), obtained by UHPLC-ESI$^+$-Orbitrap-MS; **Figure S4.** Spectra obtained by $^1$H NMR (400.144 MHz, $CD_3OD$) and by $^{13}$C NMR (100.626 MHz, $CD_3OD$) of the compound (b): (*E*)-3-(4-(((*E*)-3-(3,4-dihydroxyphenyl)acryloyl)oxy)-3-hydroxyphenyl)acrylic acid, respectively; **Figure S5**. 2D NMR Spectra HSQC and HMBC of the compound (b): (*E*)-3-(4-(((*E*)-3-(3,4-dihydroxyphenyl)acryloyl)oxy)-3-hydroxyphenyl)acrylic acid; **Figure S6.** IR spectrum of the compound (b): (E)-3-(4-(((E)-3-(3,4-dihydroxyphenyl)acryloyl)oxy)-3-hydroxyphenyl)acrylic acid; **Figure S7.** Spectra obtained by $^1$H NMR (400.144 MHz, $CD_3OD$), $^{13}$C NMR (100.626 MHz, $CD_3OD$) and Dept-135 of the compound (c): diglucosylated ursolic acid; **Figure S8**. 2D NMR Spectra HSQC and HMBC of the compound (b): diglucosylated ursolic acid; **Figure S9.** IR spectrum of the compound (b): diglucosylated ursolic acid; **Table S1.** Taxonomic identification; **Figure S10.** Chromatographic profile and retention times obtained by HPLC-DAD of certified reference phenolic compounds; **Figure S11.** Ultrasound-assisted, exhaustive extraction steps. In purple, liquid-liquid extraction. In green, solid-liquid extraction.

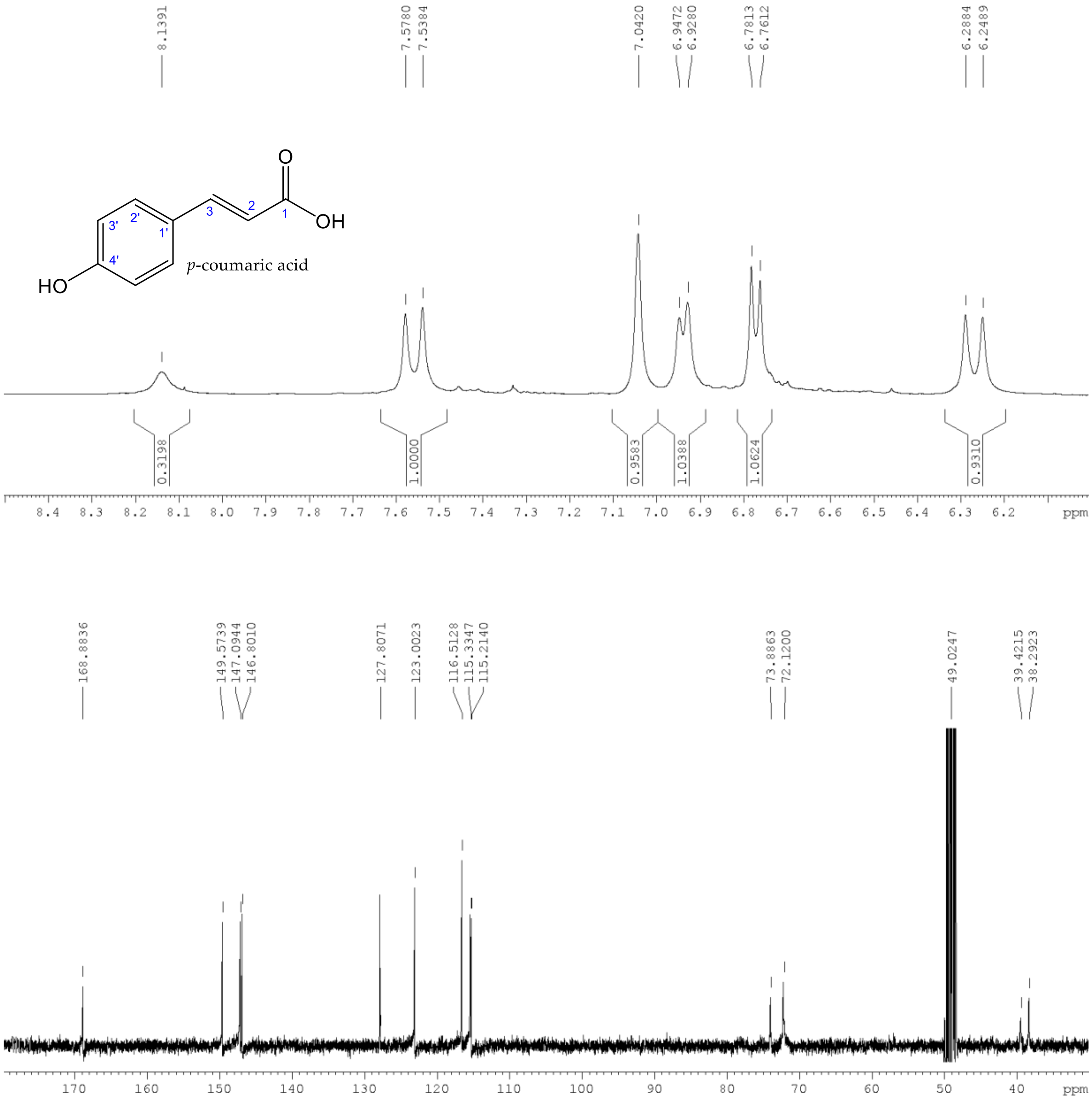


**Figure S1**. Spectra obtained by $^{1}H$ NMR (400.144 MHz, $CD_3OD$) and by $^{13}C$ NMR (100.626 MHz, $CD_3OD$) of the compound (a): *p*-coumaric acid.

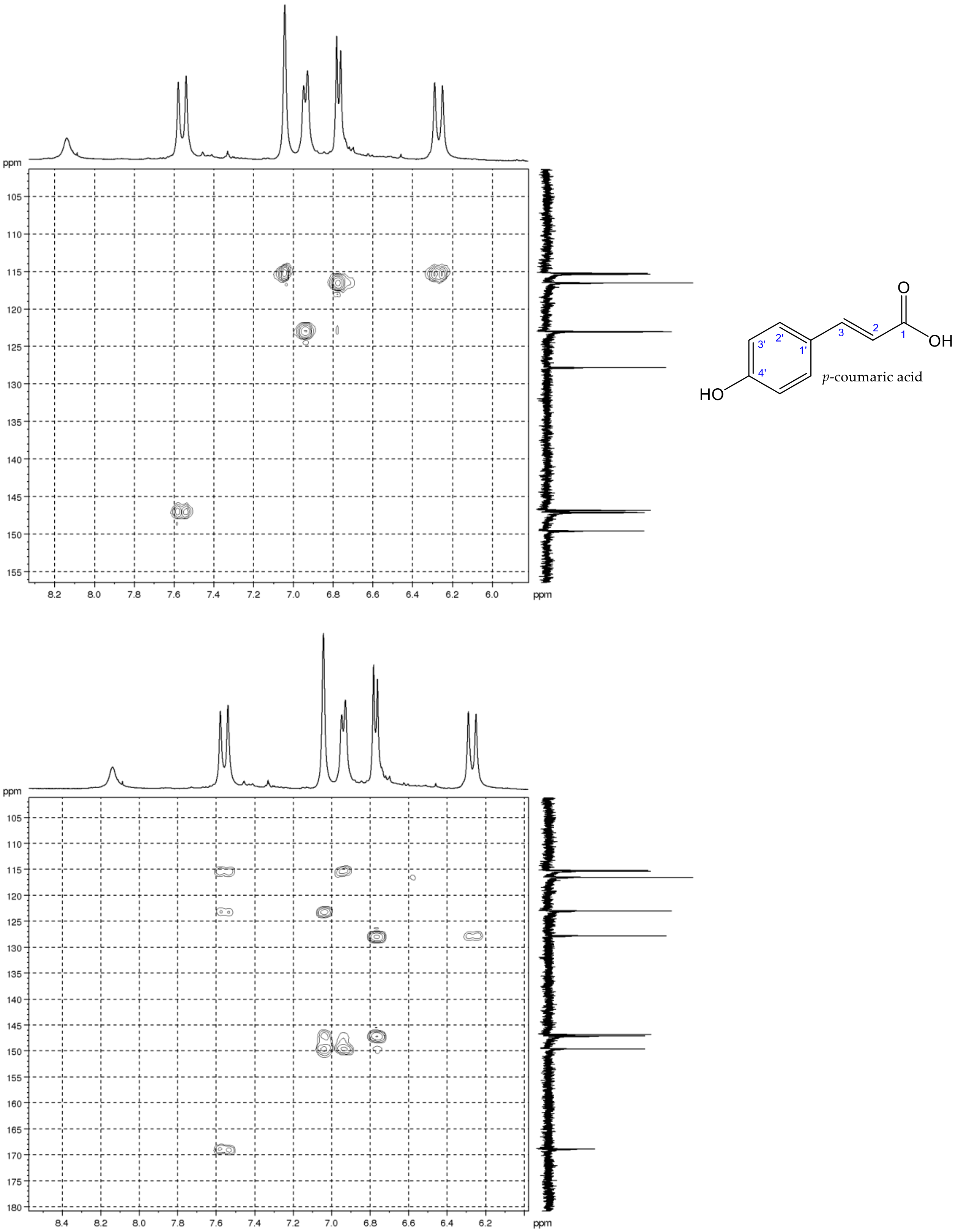


**Figure S2**. 2D NMR Spectra HSQC and HMBC of the compound (a): *p*-coumaric acid.

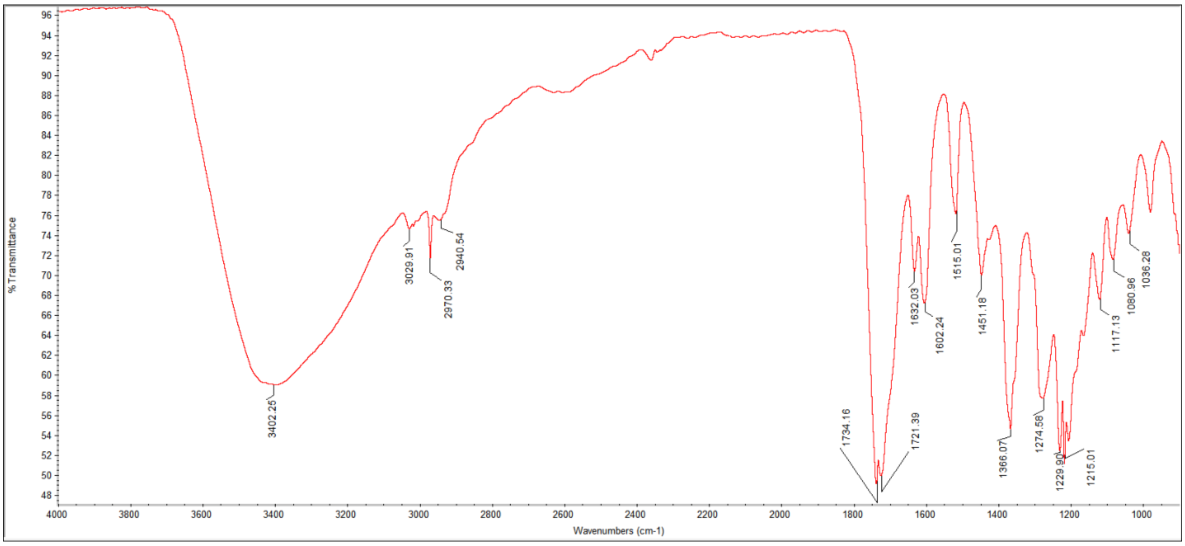


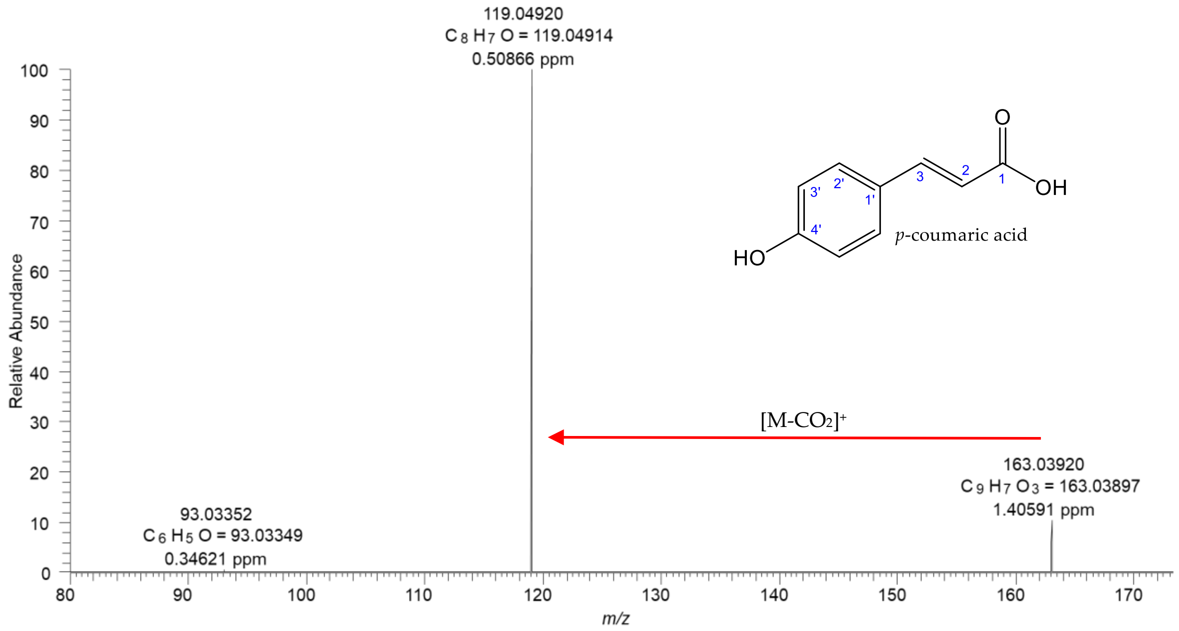


**Figure S3**: IR spectrum and Mass spectra of (a): *p*-coumaric acid (HCD, 10 eV), obtained by UHPLC-ESI$^+$-Orbitrap-MS.

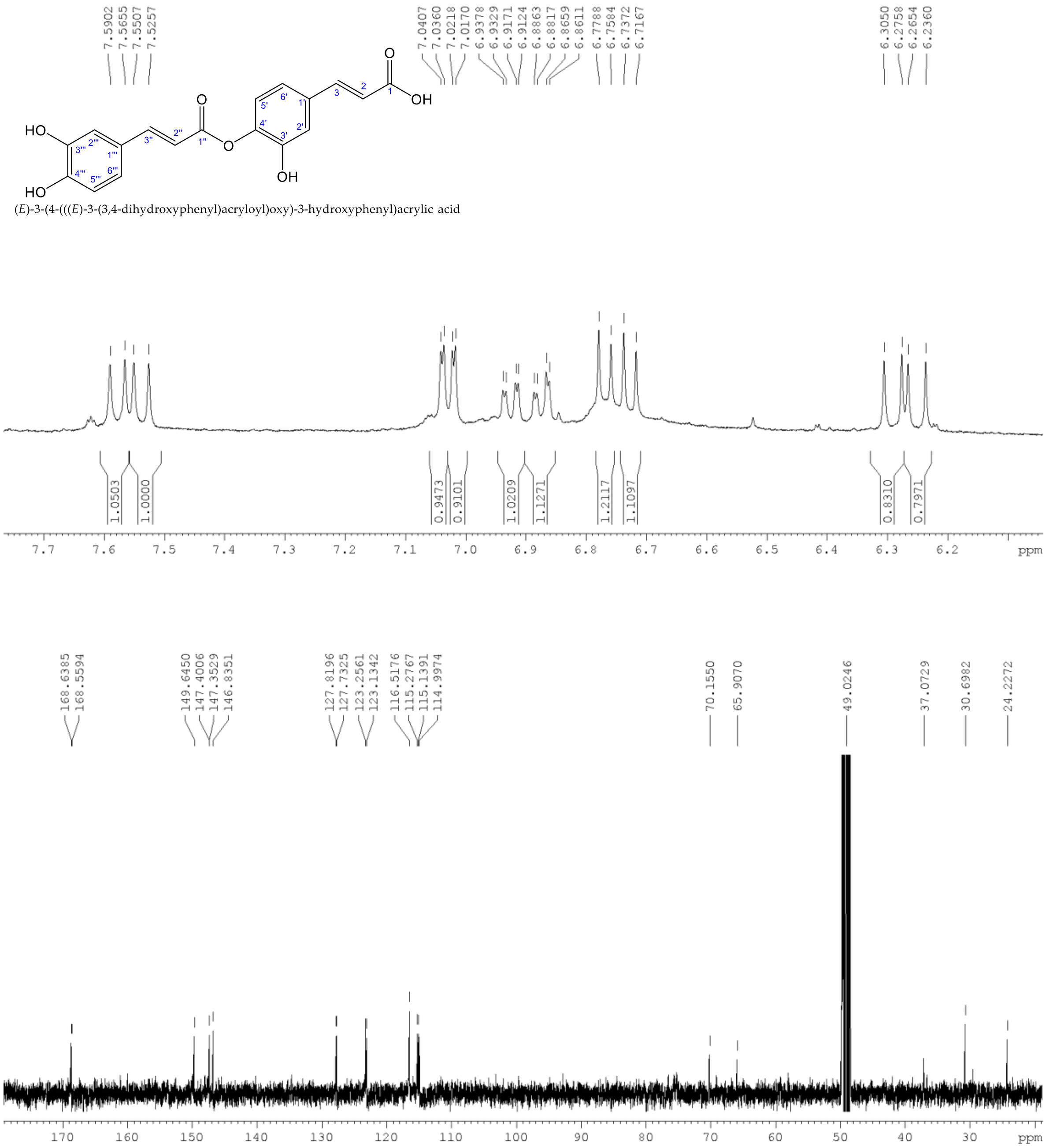


**Figure S4.** Spectra obtained by $^{1}$H NMR (400.144 MHz, $CD_3OD$) and by $^{13}$C NMR (100.626 MHz, $CD_3OD$) of the compound (b): (*E*)-3-(4-(((*E*)-3-(3,4-dihydroxyphenyl)acryloyl)oxy)-3-hydroxyphenyl)acrylic acid, respectively.

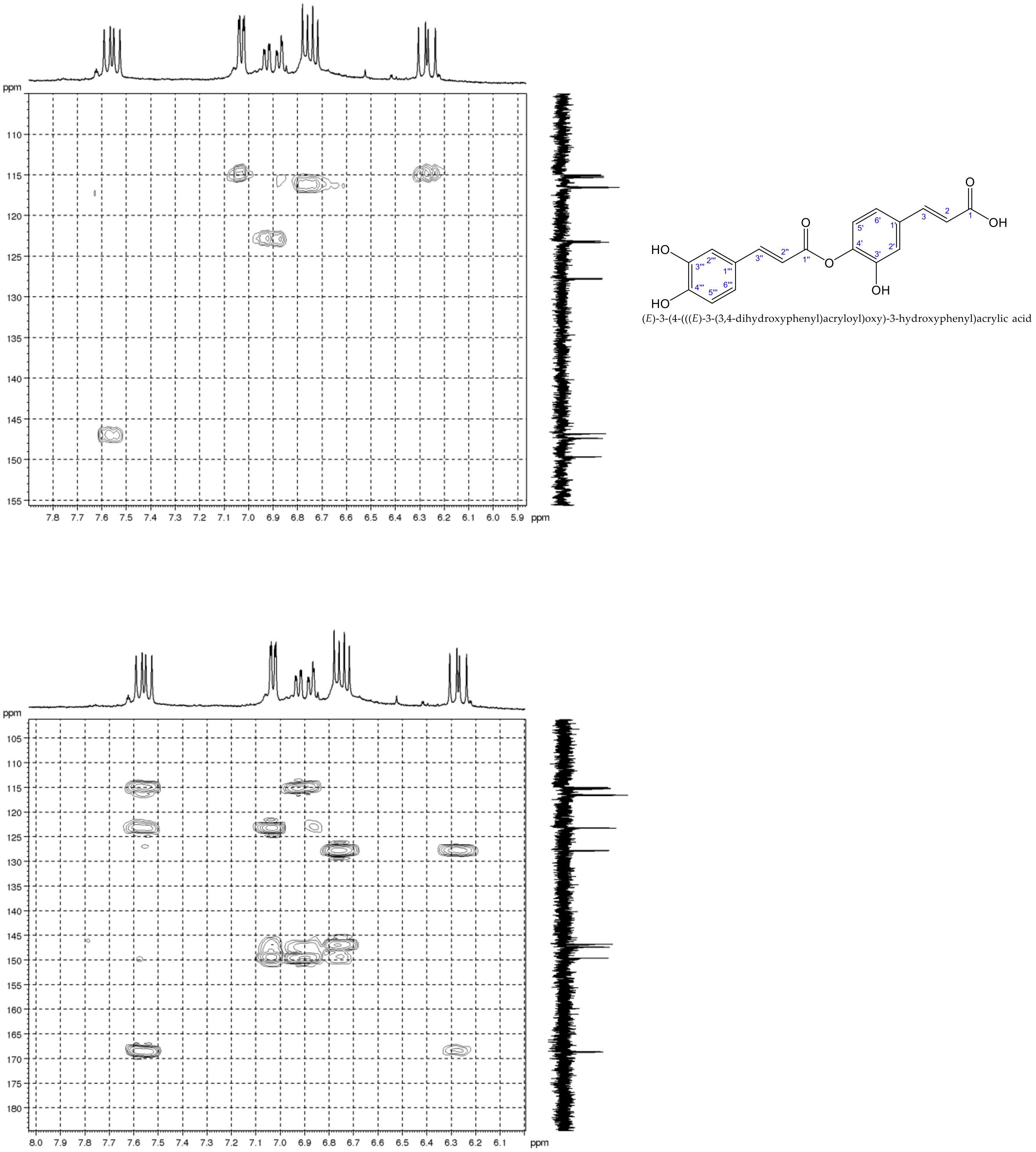


**Figure S5**. 2D NMR Spectra HSQC and HMBC of the compound (b): (*E*)-3-(4-(((*E*)-3-(3,4-dihydroxyphenyl)acryloyl)oxy)-3-hydroxyphenyl)acrylic acid.

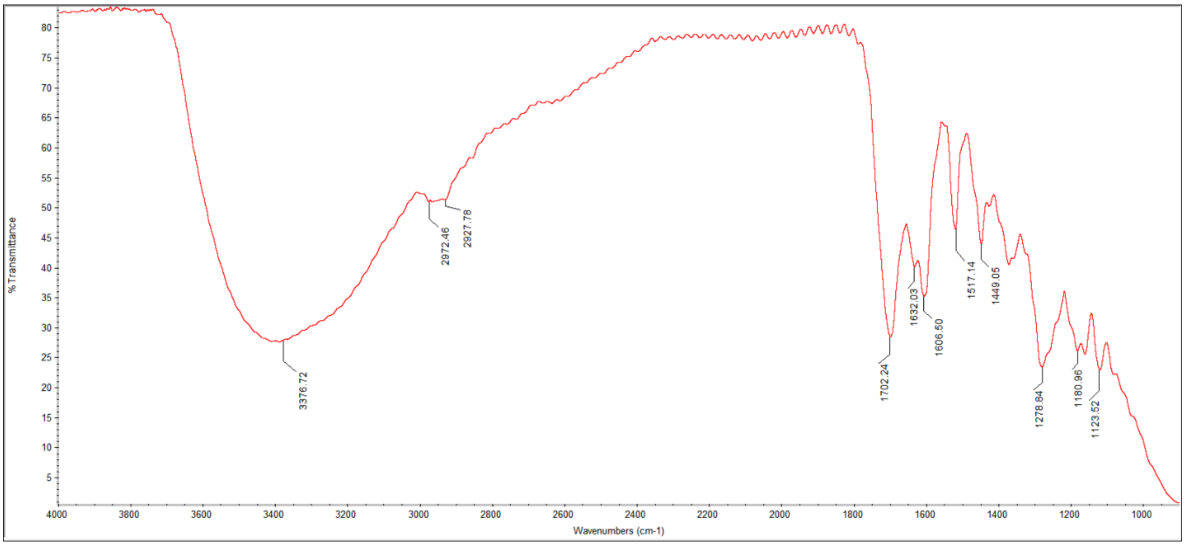


**Figure S6.** IR spectrum of the compound (b): *(E)*-3-(4-(((E)-3-(3,4-dihydroxyphenyl)acryloyl)oxy)-3-hydroxyphenyl)acrylic acid

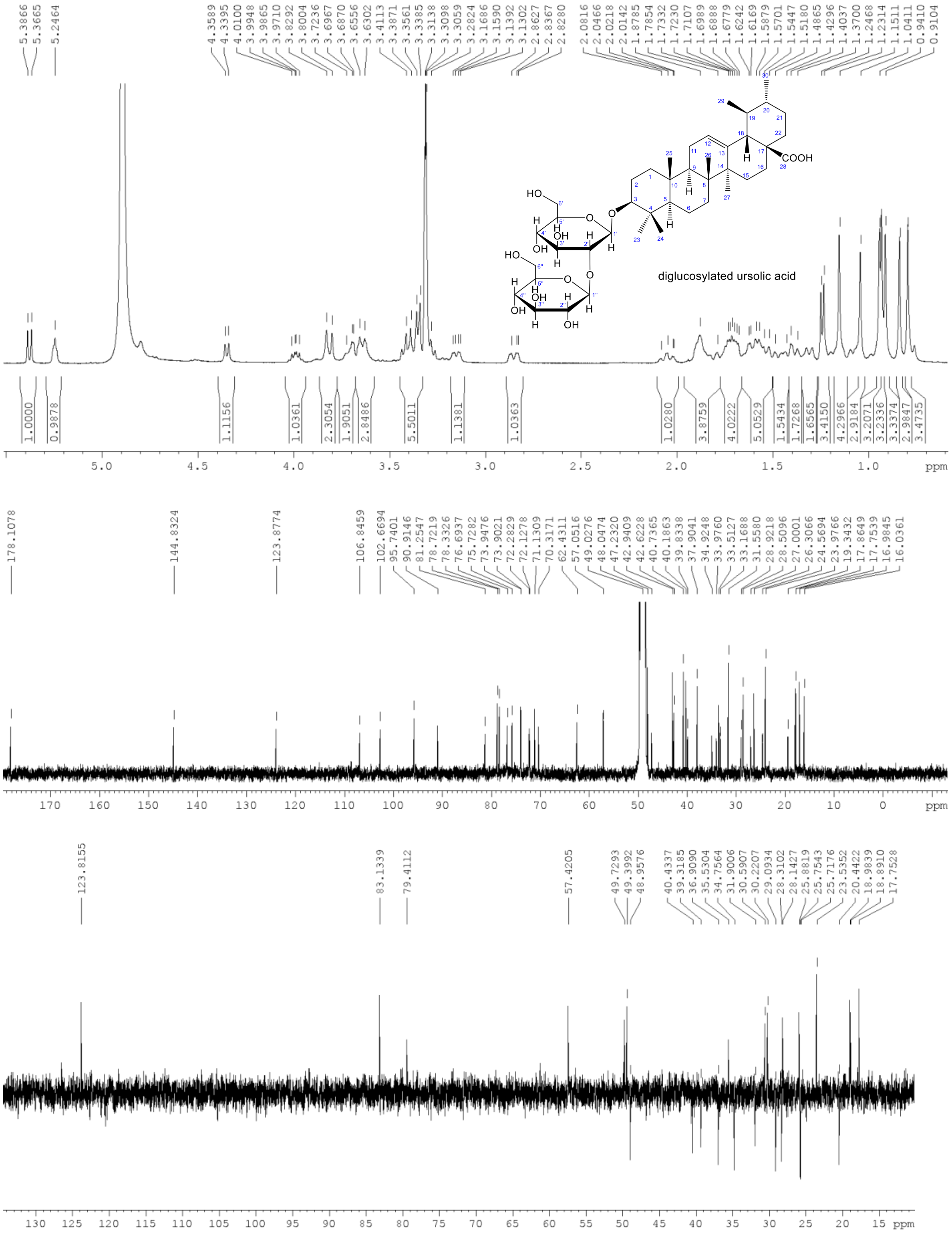

**Figure S7.** Spectra obtained by $^{1}$H NMR (400.144 MHz, $CD_3OD$), $^{13}$C NMR (100.626 MHz, $CD_3OD$) and Dept-135 of the compound (c): diglucosylated ursolic acid.

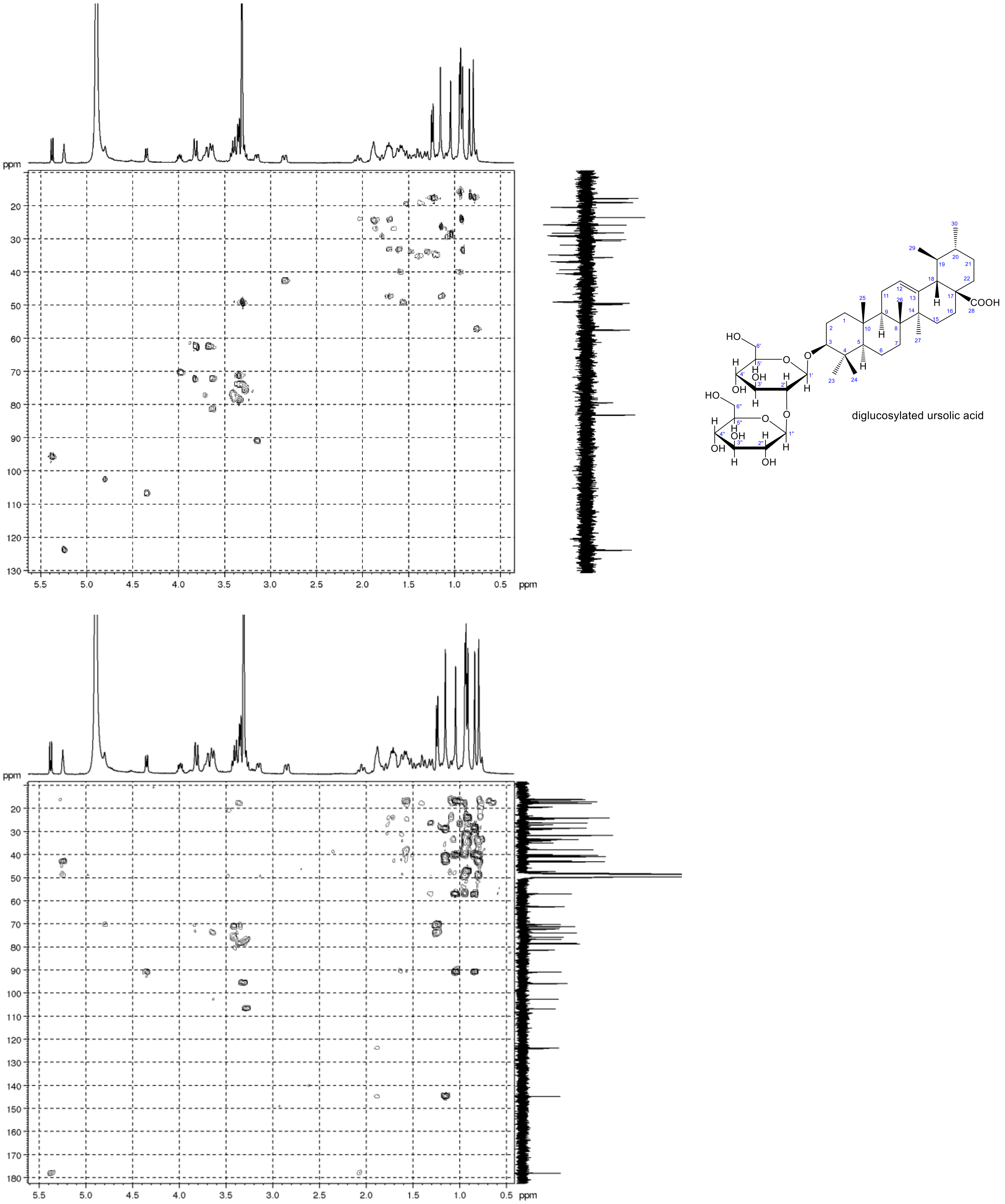


**Figure S8**. 2D NMR Spectra HSQC and HMBC of the compound (b): diglucosylated ursolic acid.

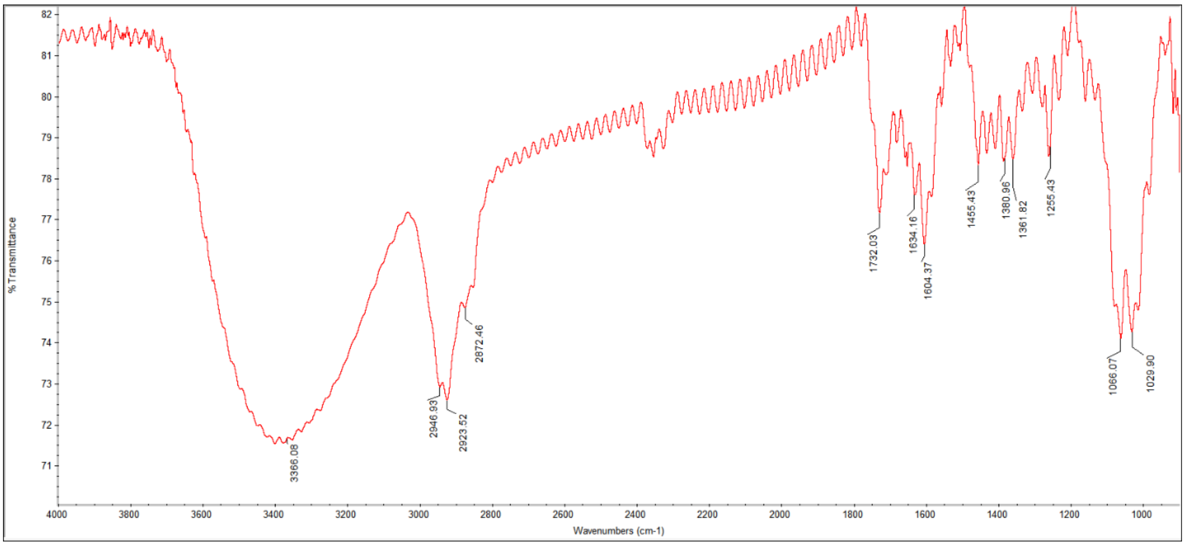


**Figure S9.** IR spectrum of the compound (b): diglucosylated ursolic acid

**Table S1.** Taxonomic identification.

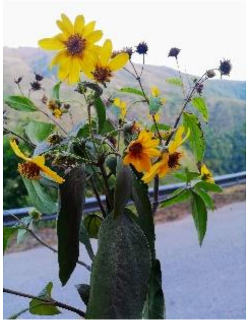

| | |
|---|---|
| Kingdom | Planatae |
| Division | Magnoliophyta |
| Class | Magnoliopsida |
| Order | Asterales |
| Family | Asteraceae |
| Genus | ***Steiractinia*** |
| Species | ***aspera*** Cuatrec |

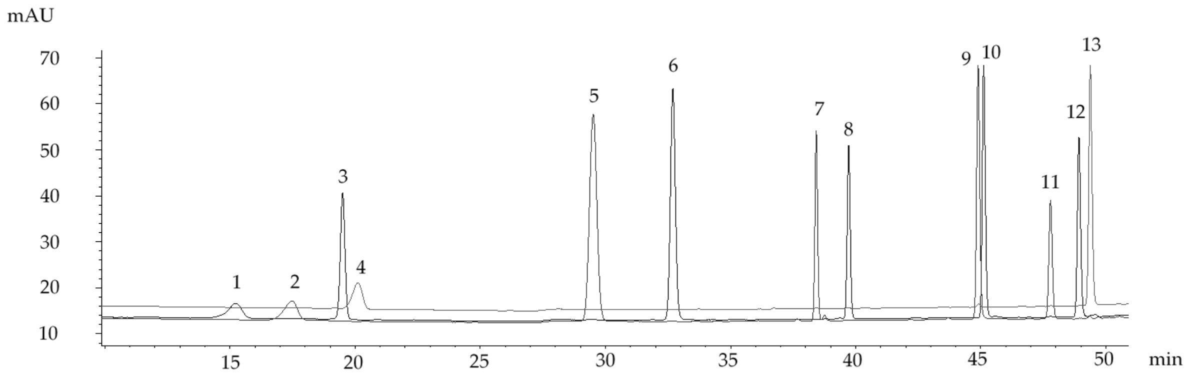


| No. peak | $t_R$ (λ=290 nm), min | Compound |
|---|---|---|
| **1** | 15,2 | Cyanidin-3,5-diglucoside |
| **2** | 17,5 | Delphinidin-3-glucoside |
| **3** | 19,5 | Chlorogenic acid |
| **4** | 20,1 | Cyanidin-3-ruthinoside |
| **5** | 29,5 | *p*-Coumaric acid |
| **6** | 32,7 | Ferulic acid |
| **7** | 38,4 | Kaempferol-3-glucoside |
| **8** | 39,7 | Rosmarinic aci |
| **9** | 44,9 | Quercetin |
| **10** | 45,1 | Luteolin |
| **11** | 47,8 | Naringenin |
| **12** | 48,9 | Apigenin |
| **13** | 49,4 | Kaempferol |

**Figure S10.** Chromatographic profile and retention times obtained by HPLC-DAD of certified reference phenolic compounds.

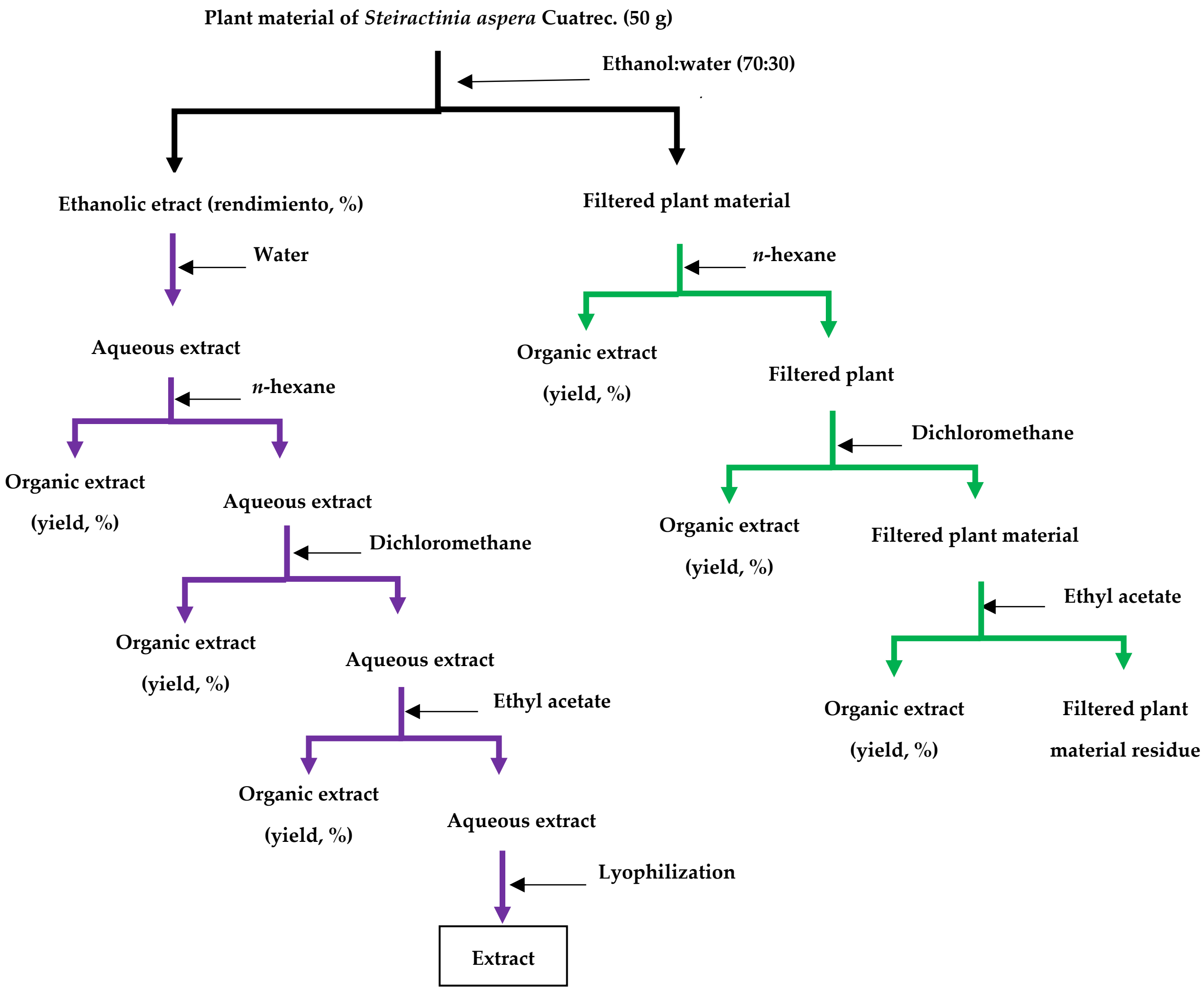


**Figure S11.** Ultrasound-assisted, exhaustive extraction steps. In purple, liquid-liquid extraction. In green, solid-liquid extraction.
